# Management of medical emergencies in orthodontic practice

**DOI:** 10.1186/s40510-020-00327-3

**Published:** 2020-08-10

**Authors:** M. Amiri Bavandpour, C. Livas, R. E. G. Jonkman

**Affiliations:** Department of Orthodontics, Academic Center for Dentistry Amsterdam (ACTA), University of Amsterdam and VU University Amsterdam, Amsterdam, The Netherlands

**Keywords:** Medical emergencies, Orthodontic practice management, Continuing professional education, Self-perception, Survey

## Abstract

**Background:**

The aims of this study were to survey the occurrence of acute medical situations in Dutch orthodontic practice and to examine the self-perceived competence of orthodontists in managing acute medical emergencies.

**Methods:**

A self-designed questionnaire was distributed among all 149 Dutch orthodontists attending the spring meeting of the Dutch Society for Orthodontists. The questionnaire was divided into three parts, addressing background information of the orthodontist, precautions against and experiences in acute medical situations, and self-perceived competence of nine common medical emergencies. The statistical analysis was performed using the chi-square test and a multiple logistic regression analysis.

**Results:**

The response rate was 74.5% (105 out of 149). The male to female ratio in this population was 55:50. Mean age of all participants was 46.9 years (SD 10.4 years) with on average 16.7 years of working experience (SD 10.6 years). The most common medical emergency reported by Dutch orthodontists was vasovagal collapse (*n* = 219), followed by acute allergic reaction (*n* = 163) and hyperventilation (*n* = 83). On average, 75% (*n* = 79) of the orthodontists felt competent to handle any acute medical situation with an average occurrence of 0.36 acute medical emergencies per orthodontist per year. Male participants were more likely to send patients towards the emergency department (*p* = 0.049). Moreover, a statistically significant negative correlation was observed between self-perceived competence handling hypoglycemia and years of clinical experience. The longer orthodontists were clinically active, the less competent they felt when encountered with a hypoglycemia (*p* = 0.031).

**Conclusions:**

Medical emergencies may be rare but challenging occurrences in the orthodontic practice. It is strongly recommended for all orthodontists and supporting staff to be trained regularly in the management of medical emergencies and to possess up-to-date evidence-based knowledge. Familiarity with and availability of appropriate drugs and equipment are deemed essential to the management of acute medical emergencies that may arise in the orthodontic practice.

## Background

Nowadays, patient safety and risk management have become a priority in dental and orthodontic practice. In Japan, 19 to 44% of the dentists experience a medical emergency in each year with the vast majority of complications occurring between patients from the age of 20 to 49 years of age [1]. A Dutch study reported an occurrence of 0.75 medical emergencies per dentist per year [2]. In a scoping review on medical emergencies in dental practice, it is reported that 43.6 to 75% of dentists will be required to perform some form of medical emergency management throughout their career [3]. Although life-threatening medical emergencies are rare in dental practice, according to Collange et al., approximately 5% of general practitioners have to cope with cardiopulmonary resuscitation (CPR) at least once during their career [4]. Members of the dental and orthodontic team are expected to adequately manage common acute medical emergencies that might occur. Nevertheless, dentists still lack skills for managing medical emergencies regardless of the incorporation of medical training into dental curricula [3, 5, 6]. Moreover, only 69% of dentists feel comfortable managing medical emergencies in a dental setting [7].

While orthodontic services have traditionally been provided to children, adolescents, and young adults, over the past few decades the population in orthodontic practice has been changing. Increasing numbers of adults are choosing orthodontic treatment [8, 9]. An older population results in more medically compromised patients due to chronic medical conditions and increased usage of long-term medication [10–14]. With the increased number of medically compromised individuals, it is imperative that orthodontists are able to manage medical emergencies [7]. The key finding of the scoping review of Vaughan et al. [3] is that there is a lack of preparedness towards medical emergencies, despite a universal recognition of the importance and desire to improve medical skills. While serious or major medical emergencies are not common in dental or orthodontic practices, being prepared to manage a medical emergency is critical, as it may in fact be life-saving [7].

To the best of the authors’ knowledge and according to the literature, no studies have been conducted on the occurrence of medical emergencies and orthodontists’ competence to cope with medical emergencies in orthodontic practice in the Netherlands. The aim of this study was to determine the occurrence of common acute medical situations within Dutch orthodontic practice and to determine the self-perceived competence of Dutch orthodontists to act adequately when encountered with certain acute medical situations.

## Methods

This survey was conducted during the Annual Spring Meeting of the Dutch Society of Orthodontists (NVvO [Nederlandse Vereniging van Orthodontisten]). At this meeting, held on the 28th and 29th of March 2019 in Soestduinen, Utrecht, the Netherlands, a questionnaire was handed out to all present orthodontists (*N* = 149). One month prior to the meeting, all participating members were informed about the research and its objectives via e-mail. The survey instrument was pre-tested and partially based on the questionnaire used by van Diermen et al. [15]. All questions were structured in a closed answer format. The questionnaire consisted of three parts. The first part addressed background information of the orthodontists including age, gender, years of practice, working affiliation, and origin of specialty training. The second part consisted of nine closed-ended questions regarding precautions against general acute medical situations and experiences in their orthodontic practice. The third part of the questionnaire was based on nine common medical emergencies seen in Dental practice, which included vasovagal collapse, hyperventilation, angina pectoris (AP), myocardial infarct (MI), cardiac arrest, aspiration, asthma, hypoglycemia, epilepsy, and an acute allergic reaction [16–18]. For all kind of emergencies, questions were asked about the occurrence of the emergency and about their self-perceived competence handling these acute medical situations when encountered in the orthodontic practice. Incomplete questionnaires and questionnaires from clinically inactive orthodontists were excluded from analysis.

### Statistical analysis

Statistical Package for Social Sciences version 25.0 and was used for data analysis. Means and standard deviations were used for descriptive statistics of all continuous variables. The chi-square test was used for 2-by-2 cross tables, and when necessary, the Fisher exact test was applied. Multiple logistic regression analysis was conducted. A significance level of *α* = 0.05 was used for all tests.

## Results

Of all questionnaires handed out, 111 were returned anonymously (response rate 74.5%). Five orthodontists did not fill in the questionnaire completely, and one orthodontist was not clinically active. The data on which the analysis was performed is based on the remaining 105 fully completed questionnaires. The male to female ratio of the population was 55:50 (52.4% male). Mean age of all participants was 46.9 years (SD 10.4 years) within the range of 31 to 72 years (all clinically active). The average years of working experience was 16.7 years (SD 10.6 years), ranging from 2 to 41 years of experience. Table 1 gives an overview by gender of age, years of professional experience, place of postgraduate training, and professional setting. Almost one third of all participants (*n* = 29/27.6%) was trained abroad.

**Table 1:** Sample characteristics

*Study sample* *n = 105*	**Male, *n*= 55 (52.4%)**				**Female, *n*= 50 (47.6%)**			
	Mean	SD	Min.	Max.	Mean	SD	Min.	Max.
Age (y)	50.6	10.1	31	72	42.7	9.1	31	61
Experience (y)	20.0	10.9	3	41	13.1	9.0	2	33
*Professional setting*		**Male,*n*(%)**				**Female,*n*(%)**		
Practice owner		40 (72.7%)				26 (52%)		
Practice owner and locum		1 (1.8%)				2 (4%)		
Practice owner and academic		4 (7.2%)				3 (6%)		
Locum		8 (14.5%)				18 (36%)		
Locum and academic		1 (1.8%)				0		
Academic		1 (1.8%)				1 (2%)		
*Orthodontic training location*		**Male,*n*(%)**				**Female,*n*(%)**		
Amsterdam		16 (29.1%)				21 (42%)		
Nijmegen		12 (21.8%)				13 (26%)		
Groningen		7 (12.7%)				7 (14%)		
Abroad		20 (36.4%)				9 (18%)		

Overview of sample size, age, years of experience, professional setting, and where the training of the orthodontists is by gender

*y* years

**Fig. 1:**
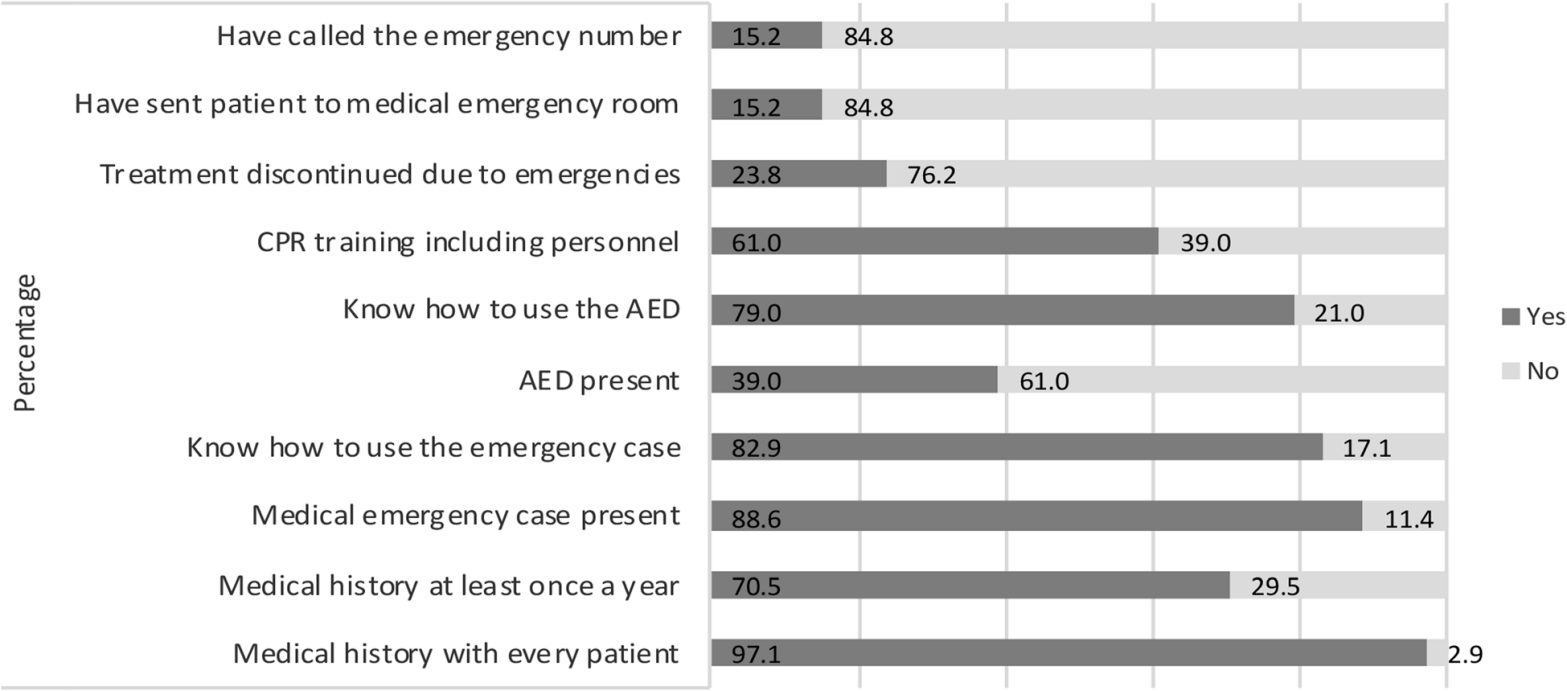
Precautions against and experiences in acute medical emergencies in orthodontic practice

Figure 1 shows the second part of the questionnaire regarding precautions against acute medical situations and past experiences with acute medical situations in orthodontic practice. Of all orthodontists, 97.1% takes a medical history before treatment and 70.5% repeats a medical history at least once a year for each patient. In 88.6% of the orthodontic practices, a medical emergency kit is present. When present, 82.9% of the orthodontists knew how to use it. In 39% of the practices, an AED (Automatic External Defibrillator) was present. Although only present in about one third of the practices, 79.0% of the participants knew how to use it. Furthermore, 61.0% of the orthodontists were CPR trained. Finally, approximately one in four orthodontists (23.8%) has discontinued treatment because of an acute medical situation at least once during their career. An even smaller number, 15.2% of all orthodontists have called the emergency line and/or have sent a patient to the emergency department of the hospital at least once during their career. For all questions, no statistical difference was found between the male and female participants (data not shown), except for the last question (*p* = 0.049). Male participants of the study have sent patients towards the emergency department of the hospital more often than the female participants (male-to-female ratio = 12:4).

Orthodontists’ experience with acute medical situations and their self-perceived competence is displayed in Table 2. On average, 75% of the orthodontists felt competent to handle any acute medical situation with an average occurrence of 0.36 acute medical emergencies per orthodontist per year. Furthermore, in most cases when an orthodontist had encountered an acute medical situation in the past, he or she felt more competent to handle this situation in the future. A statistically significant correlation was observed for aspiration (*p* = 0.025), asthmatic attack (*p* = 0.043), hypoglycemia (*p* = 0.001), epileptic attack (*p* = 0.011), and allergic reaction (*p* = 0.058). In case of an ischemic heart disease, the opposite was true. Orthodontists exposed to this acute medical situation felt less competent compared to orthodontists who had never encountered such an incident (*p* = 0.375). A cardiac arrest was never encountered in clinical practice by any of the orthodontists included in this study.

**Table 2:** Experience and perceived competence with common medical emergencies

	**Experience**	**Competence**	**Total occurrence**	**Occurrence per orthodontist per year**
Vasovagal collapse	69.5%	87.6%	219	0.125
Hyperventilation	36.2%	94.3%	83	0.047
AP/MI	4.8%	53.3%	5	0.003
Cardiac arrest	0.0%	76.2%	0,0	0,000
Aspiration	20.0%	70.5%	27	0.015
Asthma	4.8%	56.2%	12	0.007
Hypoglycemia	26.7%	76.2%	64	0.036
Epileptic insult	25.7%	84.8%	50	0.028
Allergic reaction	31.4%	76.2%	163	0.093
Total/average		75.0%	573	0,355

Percentages of orthodontists that experienced an acute medical situation (*Experience*) and that felt competent to handle a specific acute medical situation (*Competence*) are displayed. *Total occurrence* describes the number of medical emergencies that occurred during the career of all 106 orthodontists with a total of 1786 years of experience, resulting in an *occurrence per orthodontist per year*

Table 3 displays results from the multiple logistic regression analysis. A positive correlation was observed between self-perceived competence handling an aspiration and orthodontic training location. Orthodontists trained in Nijmegen felt more competent to act in case of an aspiration compared to orthodontists trained in Amsterdam (*p* = 0.023). A statistically significant negative correlation was observed between self-perceived competence handling hypoglycemia and years of clinical experience. The longer the orthodontists were clinically active, the less competent they felt when encountered with a hypoglycemia (*p* = 0.031). A tendency was observed in which women were less likely to feel competent in case of a hypoglycemia compared to men. This was also true for orthodontists trained in Groningen or abroad compared to orthodontists trained in Amsterdam. Although these correlations were not significant, a tendency was also observed in which women felt less competent compared to men in handling epilepsy (*p* = 0.065). Finally, a positive correlation tendency was observed for self-perceived competence in handling vasovagal collapse and years of clinical experience. The longer the orthodontists were clinically active, the more competent they felt in handling a vasovagal collapse, although not statistically significant (*p* = 0.070).

**Table 3:** Multiple logistic regression analysis was performed for all acute medical situations and the following predicting variables: sex, years clinically active, and training location

**Acute medical situation**	**Predicting variable**	**Coefficient**	**Standard error**	**Wald’s*χ*2**	**df**	***P*value**	**Odds ratio**	**Odds ratio 95% CI**
Epilepsy	Women	− 1.179	0.639	3.407	1	0.065	0.308	0.088–1.076
Allergic reaction	Nijmegen	1.251	0.717	3.407	1	0.081	3.494	0.858–14.236
Diabetes	Women	− 1.079	0.556	3.769	1	0.052	0.340	0.114–1.010
	Active years	− 0.055	0.026	4.628	1	**0.031**	0.946	0.900–0.995
	Groningen	− 1.518	0.784	3.745	1	0.053	0.219	0.047–1.020
	Abroad	− 1.131	0.645	3.073	1	0.080	0.323	0.091–1.143
Aspiration	Nijmegen	1.605	0.705	5.179	1	**0.023**	4.980	1.250–19.847
Vasovagal collapse	Active years	0.076	0.042	3.274	1	0.070	1.078	0.994–1.170

A significance level of *α* = 0.05 was used

*Df* degrees of freedom

## Discussion

Although medical emergencies might be rare, they are definitely challenging in the orthodontic practice. Having a medical history taken by orthodontists is an important step in preparing for a possible medical emergency and gives a better chance in preventing it [19]. In our study, 97.1% of all orthodontists take a medical history with every patient, which is outstanding though not perfect, as still 2.9% does not. Moreover, only 70.5% of the orthodontists update the medical history every year, which causes concern. It is absolutely indispensable to keep the medical history up to date. An updated medical history is of absolute necessity, and failing to obtain it is considered negligence of duty [19]. Moreover, in the Netherlands, orthodontists have an obligation to update the medical history annually in context of five annual re-registration [20].

A rather high percentage (88.6%) of the orthodontic practices has a medical emergency kit present, and 82.9% of the orthodontists also know how to use it. This is comparable to other studies where similar percentages have been reported [6, 19]. Also, the presence of a medical emergency kit is obligatory in regard to legal re-registration. This contradicts with the fact that in only 39% of the practices an AED was present, which is a very low percentage, though a rather high percentage of the orthodontists (79%) knew how to use it. Of the questioned orthodontists in this study 76.2% perceived themselves to be competent handling a cardiac arrest although only 61% of the questioned orthodontists were CPR trained. Al-Iryani et al. [19] reported that 95% of the questioned practitioners were trained in basic life support. Alhamad et al. [21] and Arsati et al. [22] reported that respectively 44.8% and 43% were skilled in performing CPR. It is at least remarkable that many orthodontists prepare themselves for handling a cardiac arrest, although cardiac arrest was never encountered by any of the participants. This contradicts with the article of Collange et al. [4] which reported that 5% of general dental practitioners will be confronted with CPR in clinical dental setting. Nevertheless, orthodontists should be trained in CPR, but also in handling less severe and more frequently occurring medical emergencies.

In this study, the most common medical emergency reported by Dutch orthodontists was vasovagal collapse, which is consistent with other studies [1, 22–26]. In contrast with literature, acute allergic reaction was the second most commonly reported emergency in our study, whereas in other studies hyperventilation, hypoglycemia and epileptic insult are reported as the second most common emergencies [1, 3, 21]. The third most reported medical emergency was hyperventilation. A minor part (4.8%) of the orthodontists encountered angina pectoris and/or a myocardial infarct. However, approximately one quarter of the orthodontists had witnessed aspiration, hypoglycemia, and an epileptic insult. It is therefore of absolute necessity that orthodontists are sufficiently trained in managing these medical emergencies.

Finally, self-perceived competence in handling acute medical situations was also evaluated in this study. Percentages ranged from 53.3% in case of angina pectoris and/ or myocardial infarction to 94.3% in case of hyperventilation. On average, 75% of the orthodontists felt competent to handle acute medical situations in orthodontic practice, which is a rather high percentage compared to literature [19]. However, still one quarter of the orthodontists, which is a considerable part, feel themselves incompetent in handling medical emergencies in orthodontic practice. It appeared that orthodontists felt more competent when he or she had been exposed to the medical emergency in the past. This information pledges for practical training. After all, when exposed to medical emergencies, in daily practice or in simulated training circumstances, orthodontists will consider themselves more competent. For ischemic heart diseases, the opposite was true, indicating possible difficulties when being confronted with this particular acute medical situation.

Interestingly, orthodontists trained in Nijmegen felt more competent to act in case of an aspiration, and orthodontists trained in Groningen or abroad were less likely to feel competent in case of a hypoglycemia compared to orthodontists trained in Amsterdam. A difference in medical training during the orthodontic traineeship may underlie this difference. Furthermore, the longer the orthodontists were clinically active, the less competent they felt when encountered with a hypoglycemia. Therefore, one might think that older orthodontists did not keep their medical knowledge up to date during their career. Although not significant, the opposite was true for vasovagal collapse, in which the longer the orthodontists were clinically active, the more competent they felt in handling a vasovagal collapse. Vasovagal collapse was the most frequently occurring acute situation in daily practice and is relatively easy to handle. Finally, a tendency was observed in which women were less likely to feel competent in case of a hypoglycemia and epilepsy compared to men (both not significant). This tendency might be explained by the difference between men and women, in which men consistently report higher self-esteem and confidence than women [27].

To the authors’ knowledge, this is the first study that reports on occurrence of medical emergencies in the orthodontic practice in the Netherlands. A strength of the study is the relatively high response rate calculated as a percentage of all registered orthodontists in the Netherlands (33.9%).

There were also several limitations to this study. First of all, this cross-sectional study relied on the memory of orthodontists, which is retrospective and might be less reliable. A limitation of the questionnaire was that no distinction was made in acute allergic reactions and delayed allergic reactions. Most probably a major part of the reported allergic reactions can be considered as local and mild and would not be considered an acute medical emergency. Also, no distinction was made between aspiration within the orthodontic practice and aspiration at home. Another limitation is the closed-ended questions that were used in the questionnaire. Participants’ self-perceived competence would be more accurately assessed with open-ended questions that provide them with the possibility to clarify and specify their answers. Furthermore, the situation in the Netherlands may be totally different from other countries, and therefore, the data cannot be extrapolated indiscriminately to other countries.

The most important question that arises from this study is whether the surveyed orthodontists were able to adequately judge their own competence. According to Lai and Teng [28], self-perceived competence does not correlate well with objectively assessed competence, although this assessment was performed in the field of general medicine. Katowa and Banda [29] observed a negative correlation between self-perceived and objectively measured competence, which demonstrates an inability of individuals to assess and rate themselves objectively. It therefore seems that people might over- or underestimate themselves in feeling competent. According to Kruger and Dunning [30], competent people can adequately judge themselves (they slightly underestimate their own performance), while incompetent people frequently overrate their own abilities. They observed that low performing participants greatly overestimated their own abilities. These individuals are especially dangerous in clinical practice and can lead to catastrophic results. One might think to be competent, but simply is unprepared in reality. A major drawback of this study therefore is that competent people were not distinguished from incompetent people, independent of their self-perceived competence. Future research on medical emergencies in orthodontic practice should therefore be based on realistic patient simulation, including the usage of emergency drugs, in which the competence of orthodontists is determined by medical professionals instead of self-perceived abilities. This could give more insight in the actual competence of orthodontists in the Netherlands regarding the management of acute medical emergencies, without the risk of over- and underestimation.

## Conclusions

- Practicing orthodontists in the Netherlands do not frequently encounter medical emergencies in daily practice, with an estimated occurrence of 0.36 acute medical emergencies per orthodontist per year. On average, 75% of the orthodontists feel competent to handle any acute medical situation.
- In general, health care providers in the Netherlands are obliged to be able to handle acute medical emergencies. It is therefore strongly recommended for all orthodontists and supporting staff to be trained regularly in the management of medical emergencies and keep their evidence-based knowledge up-to-date. Realistic patient-simulation-based training should be part of medical emergency training and annually repeated.
- Familiarity with and availability of appropriate drugs and equipment are deemed essential to the management of acute medical emergencies that may arise in the orthodontic practice.

## Supplementary information

**Additional file 1. **Questionnaire.

## Data Availability

The datasets used and/or analyzed during the current study are available from the corresponding author on reasonable request.

## Abbreviations

AED: Automatic external defibrillator
AP: Angina pectoris
CPR: Cardiopulmonary resuscitation
MI: Myocardial infarct
NVvO: Nederlandse Vereniging van Orthodontisten
