# Supplementary material for: Management of medical emergencies in orthodontic practice
Source: Prog Orthod. 2020 Aug 10;21:25. doi: 10.1186/s40510-020-00327-3 (PMC7415467; doi:10.1186/s40510-020-00327-3)
Supplement: Supplementary file 1 — Additional file 1. Questionnaire. [file 40510_2020_327_MOESM1_ESM.docx]

| **Management of medical emergencies: a survey among Dutch orthodontists** |
| --- |
| **Instruction**  This questionnaire contains six regular questions (A-F) and 36 substantive questions about the self-perceived competence and the incidence of acute medical conditions in orthodontic practice. |

| **A** | **Are you currently clinically active as an orthodontist?** | | |
| --- | --- | --- | --- |
|  | 1 | Yes | |
|  | 2 | No | ***End of the questionnaire. Please return the questionnaire.*** |

| **B** | **What is your age?**  **…………………….. years** |
| --- | --- |

| **C** | **What is your gender?** | |
| --- | --- | --- |
|  | 1 | Man |
|  | 2 | Woman |

| **D** | **In which year did you complete your training as an orthodontist?**  **………………………………..** |
| --- | --- |

| **E** | **Where did you receive your orthodontic training?** | |
| --- | --- | --- |
|  | 1 | Amsterdam |
|  | 2 | Groningen |
|  | 3 | Nijmegen |
|  | 4 | Elsewhere (please specify) ………………………………………. |

| **F** | **What is your current employment status? (multiple answers possible)** | |
| --- | --- | --- |
|  | 1 | Practice owner |
|  | 2 | Locum |
|  | 3 | Academic |

1. A. Do you take a medical history for every patient?
   1. Yes
   2. No

B. If so, do you repeat the medical history once a year?

a. Yes

b. No

1. Is a medical emergency kit present in the orthodontic practice where you are working?
   1. Yes
   2. No
2. Do you know how to apply the content of the medical emergency kit in practice?
   1. Yes
   2. No
3. Is an Automatic External Defibrillator (AED) present in the orthodontic practice where you are working?
   1. Yes
   2. No
4. Do you know how to use the AED?
   1. Yes
   2. No
5. Has cardiopulmonary resuscitation training ever taken place in your practice (including staff)?
   1. Yes
   2. No
6. Have you ever discontinued treatment because of a medical emergency?
    a. Yes

b. No

1. Have you ever referred a patient to the hospital’s emergency room because of a medical emergency?
   1. Yes
   2. No
2. Have you ever called the emergency number because of a medical emergency in your practice?
    a. Yes

b. No

**Vasovagal collaps**

1. Have you ever experienced that a patient of yours, caregiver or staff fainted in the practice where you are working or have worked?
   1. Yes
   2. No Continue to question 12
2. How often in your career have you experienced this?

…………………..

1. Do you feel competent to deal with vasovagal collapse in orthodontic practice?
   1. Yes
   2. No

**Hyperventilation**

1. Have you ever experienced that a patient of yours, caregiver or staff started to hyperventilate in the practice where you are working or have worked?
   1. Yes
   2. No Continue to question 15
2. How often in your career have you experienced this?

………………………………….

1. Do you feel competent to deal with hyperventilation in orthodontic practice?
   1. Yes
   2. No

**Angina pectoris/ Myocardial infarct**

1. Have you ever experienced that a patient of yours, caregiver or staff has developed acute cardiac chest pain in the practice where you are working or have worked?
   1. Yes
   2. No Continue to question 18
2. How often in your career have you experienced this?

……………………………

1. Do you feel competent to deal with acute cardiac chest pain in orthodontic practice?
   1. Yes
   2. No

**Cardiac arrest**

1. Have you ever experienced that a patient of yours, caregiver or staff has developed a cardiac arrest in the practice where you are working or have worked?
   1. Yes
   2. No Continue to question 21
2. How often in your career have you experienced this?

……………………………

1. Do you feel competent to resuscitate in the event of a cardiac arrest in orthodontic practice?
   1. Yes
   2. No

**Aspiration**

1. Have you ever experienced that a patient of yours, caregiver or staff has aspired a corpus alienum in the practice where you are working or have worked?
   1. Yes
   2. No Continue to question 24
2. How often in your career have you experienced this?

……………………………

1. Do you feel competent to cope with aspiration in orthodontic practice?
   1. Yes
   2. No

**Asthma**

1. Have you ever experienced that a patient of yours, caregiver or staff developed an asthmatic attack in the practice where you are working or have worked?
   1. Yes
   2. No Continue to question 27
2. How often in your career have you experienced this?

……………………………

1. Do you feel competent to cope with an asthmatic attack in orthodontic practice?
   1. Yes
   2. No

**Diabetes**

1. Have you ever experienced that a patient of yours, caregiver or staff had a hypoglycaemia in the practice where you are working or have worked?
   1. Yes
   2. No Continue to question 30
2. How often in your career have you experienced this?

……………………………

1. Do you feel competent to cope with a hypoglycaemia in orthodontic practice?
   1. Yes
   2. No

**Epilepsy**

1. Have you ever experienced an epileptic seizure of a patient of yours, caregiver or staff in the practice where you are working or have worked?
   1. Yes
   2. No Continue to question 33
2. How often in your career as an orthodontist have you experienced this?

……………………………

1. Do you feel competent to cope with an epileptic seizure in orthodontic practice?
   1. Yes
   2. No

**Allergic reaction**

1. Have you ever experienced that a patient of yours, caregiver or staff developed an allergic reaction in the practice where you are working or have worked?
   1. Yes
   2. No Continue to question 36
2. How often in your career have you experienced this?

……………………………

1. Do you feel competent to cope with an allergic reaction in orthodontic practice?
   1. Yes
   2. No
